# Relations among Romantic Myths, Offline Dating Violence Victimization and Cyber Dating Violence Victimization in Adolescents

**DOI:** 10.3390/ijerph17051551

**Published:** 2020-02-28

**Authors:** María-Jesús Cava, Sofía Buelga, Laura Carrascosa, Jessica Ortega-Barón

**Affiliations:** 1 Faculty of Psychology, Department of Social Psychology, University of Valencia, Avda. Blasco Ibáñez 21, 46010 Valencia, Spain; Sofia.Buelga@uv.es; 2 Valencian International University-VIU, Calle Pintor Sorolla 21, 46002 Valencia, Spain; laura.carrascosa@campusviu.es; 3 Faculty of Education, International University of la Rioja (UNIR), Avenida de la Paz, 137, 26006 Logroño, La Rioja, Spain; jessica.ortega@unir.net

**Keywords:** cyber dating violence, victimization, romantic myths, offline dating violence, gender analysis, adolescents

## Abstract

Cyber dating violence is an increasing problem with serious negative consequences for adolescents. Further knowledge about related variables is necessary to develop preventive strategies. The aim of this study was to analyze the correlations among cyber dating violence victimization (cyber-control and cyber-aggression), offline dating violence victimization (physical, verbal–emotional, and relational) and adolescents’ beliefs in myths of romantic love; and to examine possible differences in cyber-control victimization, cyber-aggression victimization and offline dating violence victimization (relational, physical and verbal–emotional) according to adolescents’ levels of belief (low vs. high) in myths of romantic love. The role of offline dating violence victimization (physical, verbal–emotional and relational) and romantic myths as predictor variables of cyber-control and cyber-aggression victimization was also explored. All these analyses were carried out separately with boys and girls. Of an initial sample of 919 adolescents, those who have had a dating relationship in the past year (492 adolescents, *M* = 15.10, *SD* = 1.59) were included. The regression analyses revealed that offline dating violence victimization and romantic myths were significant predictors of cyber-control and cyber-aggression victimization for both boys and girls, but explained variance was higher for girls. Verbal–emotional offline dating violence victimization was the main predictor of cyber-control victimization, and physical and relational offline dating violence victimizations were the main predictors of cyber-aggression victimization. These results can be useful for developing more effective offline and cyber dating violence prevention programs.

## 1. Introduction

Cyber dating violence is a growing problem among adolescents [1,2,3,4,5,6,7,8]. Their frequent use of the communication technologies in their romantic relationships increases the possibility of expressing affection to their partners and being linked to them [9,10]. However, these technologies can also be used to stalk, harass and control current or former partners [7,8,11,12,13,14,15]. It is estimated that between 12% and 56% of adolescents have experienced cyber dating violence victimization [16]. This form of dating violence (DV) is defined as the control, harassment, threats, stalking and abuse of current or former dating partners via technology and social media [4,5,17,18,19]. Cyber DV includes both behaviors that involve harming victims through direct attacks, e.g., threats, insults or disseminating private information, namely cyber-aggression, and forms of abusive control of victims to monitor their social relationships and what they are doing at any time, namely cyber-control [1,11,18,20,21]. Cyber-control behaviors toward partners are more frequent than cyber-aggression behaviors [20,22,23].

Cyber DV victimization is considered qualitatively different from victimization via offline dating violence because victims of such violence can be constantly attacked and feel unable to escape the situation [4,5,7,8,12]. Easy access to victims, lack of geographical boundaries and the rapid dissemination of victims’ denigrating or humiliating information are characteristics of this type of violence that make victims feel increasingly helpless, which negatively impacts their well-being [4,6,7,8,11,13,24,25,26]. Information and communication technologies (ICTs) allow prejudicial information and images about victims to quickly spread to a very large audience, constant monitoring of their activity on social networks, and contact with them anytime and from anywhere [14,19,27]. Cyber DV victimization is associated with anxiety, psychological distress, depressive symptomatology and low self-esteem in adolescents [5,6,7,14,19]. As online aggressions toward victims can be constant and humiliating online information cannot be eliminated, cyber DV victimization has been suggested to have a more negative impact on victims than offline DV victimization [14,19]. Moreover, some adolescents suffer both cyber and offline DV victimization, which increases these negative consequences [16,19]. The co-occurrence of traditional forms of DV victimization, such as physical or verbal–emotional victimization and cyber DV victimization in adolescents, has been observed in previous studies [2,6,14,16,28]. Although studies on cyber DV are still scarce, a high percentage of adolescent victims of physical and psychological–emotional DV, who are also victims of cyber DV, has been observed [6,14]. Indeed, offline DV victimization could be a risk factor for experiencing cyber DV victimization. Nowadays, a marked continuity of the real world with the virtual world exists in relationships among adolescents, and partner violence in their romantic relationships can easily move from the real world to the virtual world [22]. However, offline DV victimization includes different forms of victimization (physical, psychological, relational) that can be differently related to cyber DV victimization.

### 1.1. Offline and Cyber Dating Violence Victimization

Offline DV victimization includes more traditional forms of victimization, such as physical, relational and psychological–emotional victimization, which is experienced as part of dating relationships [29]. Physical victimization involves suffering the intentional use of physical force (e.g., hitting, pushing, slapping), relational victimization involves being victims of actions executed by the partner in order to gain control of their social relationships (e.g., demanding to end some friendship), and psychological–emotional victimization involves being victims of threats, insults or emotional blackmails. The prevalence of offline DV victimization in adolescents is high, especially the psychological–emotional victimization [30,31,32,33,34,35]. Offline DV victimization in a romantic relationship negatively affects adolescents’ well-being and has been related to stress, anxiety, less satisfaction with life, poor academic achievement, low self-esteem, substance abuse, and eating disorders [30,31,33,36,37]. Positive correlations among different forms of offline DV victimization (physical, verbal–emotional, and relational) have been observed [38], and it has also been suggested that being victimized in one social context increases the probability of being victimized in other social contexts, which could increase the possibility of suffering poly-victimization [39,40,41,42,43,44]. However, no study has yet explored if different forms of offline DV victimization, such as physical, verbal–emotional or relational victimization, have varying predictive weights in relation to distinct forms of cyber DV victimization: cyber-control and cyber-aggression. As the real world and the virtual world are closely connected in adolescents, more in-depth knowledge of these specific relations could help to develop more effective prevention strategies.

Furthermore, it is important to analyze possible gender differences in the predictive weight of the various forms of offline DV victimization in relation to cyber-control and cyber-aggression victimization. Gender differences in cyber DV victimization have been previously analyzed, but without conclusive results [14]. Some studies have shown more cyber DV victimization in girls [7,8,45], while others have indicated more cyber victimization in boys [3,46]. In studies differentiating between cyber-control and cyber-aggression, more involvement of boys in cyber-aggression behaviors and a similar involvement of boys and girls in cyber-control behaviors have been observed [22]. Research into gender differences in cyber DV victimization should be extended by analyzing not only gender differences in prevalence, but also possible gender differences in variables related to cyber DV victimization. The links between distinct forms of offline DV victimization and cyber-control and cyber-aggression victimization could differ between boys and girls.

### 1.2. Myths of Romantic Love and Cyber Dating Violence Victimization

Myths of romantic love are frequent in adolescent boys and girls, and significantly influence their perceptions of how their first romantic relationships should be [47,48]. Romantic myths include beliefs about the power of love to cope with all kinds of difficulties, perceiving love as suffering, considering jealousy to be a sign of love, the need to have a romantic love to be happy, and the existence of our soul mate who is our only one true love [48]. These romantic myths affect how adolescents initiate and maintain their romantic relationships, and what behavior they consider is normal in them [48,49,50,51]. These romantic myths can also influence adolescents’ acceptance of some abusive behavior performed by romantic partners. Not recognizing some abuse behaviors increases the probably of suffering victimization because victims are not fully aware of abusive behaviors [52]. Adolescents’ beliefs in romantic myths could make them believe that certain abusive, controlling and jealous behaviors are signs of love and are normal behaviors in a romantic relationship [39,41,42,47,53]. In line with this, some previous studies have linked offline DV victimization with myths of romantic love [47,48,50,51,54,55].

Adolescents’ beliefs in myths of romantic love might not only be related to offline DV victimization but could also increase adolescents’ risk of suffering cyber-control and cyber-aggression behaviors in their romantic relationships. Moreover, as one of the myths of romantic love includes the association of jealousy and love, the connection with cyber-control victimization could be closer. Relations between romantic myths and cyber DV victimization in young couples have been previously observed [23]. However, possible associations of cyber-control and cyber-aggression victimization with adolescents’ beliefs in romantic myths of love have barely been explored. In addition, it would be worth analyzing whether the link between DV victimization and beliefs in myths of romantic love is closer in girls given the stronger impact of these myths on girls through socialization processes and the influence of social media.

### 1.3. The Present Study

By taking into account the negative consequences of cyber DV victimization and the need to explore its relations with other variables, this study aimed to increase our knowledge about some variables related to cyber-control and cyber-aggression victimization in adolescent boys and girls. More specifically, the objectives of this study were to: (a) analyze correlations among cyber-control victimization, cyber-aggression victimization, offline DV victimization (relational, physical and verbal–emotional) and adolescents’ beliefs in myths of romantic love; (b) examine possible differences in cyber-control victimization, cyber-aggression victimization and offline DV victimization (relational, physical and verbal–emotional) according to adolescents’ levels of belief (low vs. high) in myths of romantic love; (c) analyze the role of romantic myths and offline DV victimization (physical, verbal–emotional and relational) as predictor variables of cyber-control and cyber-aggression victimization. All these analyses were performed separately with boys and girls. Regarding these objectives, the following hypotheses were proposed:

**Hypothesis** **1.** Positive correlations among all three forms of offline DV victimization (physical, verbal and relational), romantic myths, and cyber-control and cyber-aggression victimization were expected in both boys and girls. As adolescents closely link the real world and the virtual world, we expected positive correlations to appear among offline forms of DV victimization (physical, verbal and relational) and online forms of DV victimization (cyber-control and cyber-aggression). We also expected positive correlations among romantic myths and all forms of online and offline DV victimization in both boys and girls because some romantic myths include the co-existence of violence and love, the omnipotence of love to cope with all kinds of difficulties, and the perception of jealousy and control as signs of love [47,48]. Nevertheless, taking into account that romantic myths play a more important role in developing an idealized view of love in girls than in boys [48], we expected higher positive correlations among romantic myths and cyber and offline DV victimization in girls.

**Hypothesis** **2.** Adolescent boys and girls with more beliefs in myths of romantic love were expected to obtain higher scores for offline DV victimization (relational, physical and verbal) and cyber DV victimization (cyber-control and cyber-aggression). As some romantic myths involve believing in the need to have a romantic partner to be happy, thinking that true love can get over all kinds of difficulties, perceiving jealousy as a requirement of true love, and believing that love and violence can be compatible in a romantic relationship [48], we expected the adolescents who accept these myths of romantic love to more easily accept and tolerate some violent behaviors in their romantic relationships.

**Hypothesis** **3.** Romantic myths and offline DV victimization (physical, relational and verbal–emotional) will be predictors of cyber-control and cyber-aggression victimization for both boys and girls. Nevertheless, as romantic myths are more relevant for developing an ideal image of love in girls [48], romantic myths were expected to be a stronger predictor variable for cyber-control and cyber-aggression victimization in girls. Moreover, different predictive weights of the three offline DV victimization predictor variables (physical, relational and verbal–emotional) were expected for cyber-control and cyber-aggression in adolescent boys and girls.

## 2. Materials and Methods

### 2.1. Participants

This research involved adolescent boys and girls aged between 12 and 18 years old who were studying Secondary Education in three public schools in the Valencian region (eastern Spain). The reference population included adolescents studying Compulsory Secondary Education in this region. The sample size of adolescents corresponding to the student group size in Compulsory Secondary Education in the Valencian region, with a sampling error of ± 2%, a confidence level of 95%, and *p* = *q* = 0.5, (*n* = 212,285), was estimated to be 455 students. The initial sample was made up of 919 adolescents (48.1% boys, 51.9% girls; *M* age = 14.90; *SD* = 1.60). In all, 41 classes participated in this study.

Of this initial sample, only the adolescents who were in a dating relation at that time, or had been in one in the past year, were herein considered. These adolescents were asked to fill out the scale about their partner by referring to the latest relationship they had been in. Prior to encoding data, 3% of the cases were eliminated because of errors or omissions in their responses. The final sample comprised 492 adolescents, 229 boys (46.5%) and 263 girls (53.5%) aged between 12 and 18 years old. The mean age of the boys (*M* = 15.28; *SD* = 1.64) and girls (*M* = 14.94; *SD* = 1.55) was similar. The highest percentages of adolescents were aged 14 (20.4%), 15 (20.2%) and 16 years (18.6%), with lower percentages for those aged 12 (3%), 13 (15.6%), 17 (14.6%) and 18 years (7.5%). The mean age of partners was 15.79 years old (*SD* = 2.45). Regarding the length of romantic relationships, the majority of adolescents (51%) reported relationships lasting between one and six months, with lower percentages of romantic relationships lasting less than one month (17.6%), between six months and one year (14.2%), and over one year (17.2%). The romantic relationships lasting less than one month were more frequent in the adolescents aged between 12 and 15 years. Although romantic relationships are usually short in this life stage (early adolescence), they are of much emotional importance for adolescents [56].

### 2.2. Instruments

Myths of Romantic Love Scale [47,48] includes seven items that evaluate adolescents’ beliefs in myths of romantic love: soul mate (“We all have a single ideal partner, our ‘soul mate’”), jealousy as a sign of love (“When my partner controls me, he/she shows me his/her love”), omnipotence of love (“If I show him/her that I love him/her, he/she will change and make me happy”), the need to have a partner (“Separating from the couple is a failure”) and the love–violence compatibility (“You can mistreat someone you love”). Adolescents answered these items using five options ranging from 1 (strongly disagree) to 5 (strongly agree). In this sample, the reliability (Cronbach’s alpha) of this scale was 0.76.

Conflict in Adolescent Dating Relationships Inventory–CADRI [57,58] evaluates different forms of violence perpetration and victimization in adolescent couples. In this study, three forms of DV victimization (relational, physical, and verbal–emotional) were measured using three subscales of the CADRI. The Relational Victimization subscale includes three items describing situations in which adolescents’ social relationships have been negatively affected by their partners, for example being isolated from family or friends or spreading false rumors (e.g., “My partner said things to my friends about me to turn them against me”); Physical Victimization subscale comprises four items describing situations in which adolescents have experienced physical abuse from their partners, such as being pushed or hit (e.g., “My partner slapped me or pulled my hair”); and Verbal–emotional Victimization subscale includes 10 items describing situations in which adolescents have experienced emotional/psychological abuse by their partners like being insulted, threatened, or humiliated (e.g., “My partner insulted me with put-downs”). Adolescents responded to these items with four options: 1 (never), 2 (seldom: 1–2 times), 3 (sometimes: 3–5 times), and 4 (often: 6 times or more). The reliability (Cronbach’s alpha) scores of these subscales in this study were 0.72, 0.86, and 0.87, respectively.

Cyber-Violence in Adolescent Couples Scale [1] includes two subscales, Cyber-violence perpetrated and Cyber-victimization. In this study only the Cyber-victimization subscale was used, which includes 10 items about aggressive and control behaviors that romantic partners may have perpetrated against them through social media. These 10 items are integrated into two factors: Cyber-control (excessive control behaviors, e.g., “My partner doesn’t let me chat with some friends and if I do, he/she get angry”) and Cyber-aggression (threats and insults through social media; e.g., “My partner has spread malicious rumors or lies about me though social networks”). Adolescents answered these items by indicating the frequency with which their partners had performed these behaviors according to four response possibilities: 1 (never), 2 (seldom), 3 (sometimes), and 4 (often). The internal consistency (Cronbach’s alpha) of these two factors in this study was 0.83 for cyber-control and 0.88 for cyber-aggression.

### 2.3. Procedure

Three different-sized Secondary Education schools located in different areas of the Valencian region were selected. The researchers of this study contacted the selected schools. Initial contact was made by telephone to request a meeting to explain the research objectives. During this meeting, the research objectives were explained to the teachers and their collaboration was requested. Adolescents’ families were informed about the research proposal by letter, and their consent to allow their children to participate in this study was required. The parents who did not wish their children to participate indicated this to the school on a form that was attached to the information letter. They had two weeks to indicate that they did not wish their children to participate. Only 1% of the parents did not allow their children to participate. Adolescents were informed that their participation in this research was voluntary and anonymous, their data would remain confidential, and they could drop out of the study at any time. No students refused to participate. Adolescents responded anonymously without providing any identification during a regular class period (55 min). Their anonymous information was used only by the research team, who attended this session and collected the scales when students had completed them. Data collection in the participating schools lasted one month. This study was approved by the Ethics Committee of the University of Valencia (Protocol Number: H1456762885511).

### 2.4. Data Analyses

Pearson’s correlations among romantic myths, offline DV victimization (relational, verbal–emotional, and physical) and cyber DV victimization (cyber-control and cyber-aggression) were calculated separately for boys and girls. Next, the scores for offline DV victimization (relational, verbal–emotional, and physical) and cyber DV victimization (cyber-control and cyber-aggression) were compared in adolescent boys and girls with different levels (high vs. low) of belief in myths of romantic love by applying an analysis of variance (ANOVA). These two adolescent groups (high and low levels of belief in myths of romantic love) were established according to their scores for the variable romantic myths. The adolescent boys whose scores exceeded the mean score for boys in romantic myths by one standard deviation (score > 2.97; *M* = 2.22, *SD* = 0.75) were assigned to the group of high belief in romantic myths. The boys whose scores were lower than the mean score for boys by one standard deviation (score < 1.47; *M* = 2.22, *SD* = 0.75) were assigned to the group of low belief in romantic myths. Likewise, the adolescent girls whose scores exceeded the mean score for girls in romantic myths by one standard deviation (score > 2.33; *M* = 1.76, *SD* = 0.57) were assigned to the group of high belief in romantic myths. The girls whose scores were lower than the mean score for girls by one standard deviation (score < 1.19; *M* = 1.76, *SD* = 0.57) were assigned to the group of low belief in romantic myths. Finally, linear regression analyses were carried out to analyze the role of romantic myths and offline DV victimization (physical, verbal–emotional and relational) as predictor variables of cyber-control and cyber-aggression victimization. Odds ratios with a 95% confidence interval were computed by regression analyses to establish which variables were more associated with cyber-control and cyber-aggression. These linear regression analyses were carried out for boys and girls separately. All the analyses were performed with the SPSS-26 statistical package (IBM Corp., Armonk, NY, USA).

## 3. Results

### 3.1. Correlations among Variables

Table 1 shows the correlations among the variables herein included in adolescent boys and girls. The results of these analyses indicated significant positive correlations among all the forms of offline DV victimization (relational, verbal–emotional, and physical) and cyber DV victimization (cyber-control and cyber-aggression) in both boys and girls. The significant positive correlations between romantic myths and cyber DV victimization (cyber-control and cyber-aggression) were observed in boys and girls. Significant positive correlations between romantic myths and verbal–emotional and physical offline DV victimization were also observed in girls.

### 3.2. Offline and Cyber Dating Violence Victimization according to Adolescents’ Levels of Belief (Low vs. High) in Myths of Romantic Love

The analyses of possible differences in offline DV victimization (relational, verbal–emotional, and physical) and cyber DV victimization (cyber-control and cyber-aggression) in adolescent boys and girls with low and high scores for myths of romantic love are shown in Table 2. These analyses revealed significantly higher scores for verbal–emotional offline DV victimization (*F* = 6.836; *p* < 0.05; η^2^ = 0.078), cyber-control victimization (*F* = 10.211; *p* < 0.01; η^2^ = 0.112), and cyber-aggression victimization (*F* = 4.299; *p* < 0.05; η^2^ = 0.050) in adolescent girls with more beliefs in romantic myths. Adolescent boys with more beliefs in romantic myths obtained significantly higher scores for cyber-control victimization (*F* = 5.374; *p* < 0.05; η^2^ = 0.062) and cyber-aggression victimization (*F* = 4.553; *p* < 0.05; η^2^ = 0.052).

### 3.3. Romantic Myths and Offline Dating Violence Victimization as Predictors of Cyber Dating Violence Victimization

A linear regression analysis using the stepwise method was conducted separately for boys and girls, in which romantic myths and relational, verbal–emotional and physical offline DV victimization were taken as predictor variables, and cyber-control victimization was the dependent variable. Next, a similar linear regression analysis was conducted with the same predictor variables in which cyber-aggression victimization was the dependent variable. The results of these analyses are provided in Table 3 and Table 4. Regarding the variable cyber-control victimization (see Table 3), this regression model explained 33% of variance for boys, and 43% of variance for girls. For boys, the variables romantic myths (β = 0.14, *p* = 0.012) and relational and verbal–emotional offline DV victimization predicted greater cyber-control victimization (relational: β = 0.19, *p* = 0.010, verbal–emotional: β = 0.41, *p* < 0.001). For girls, the variables romantic myths (β = 0.16, *p* = 0.001), and verbal–emotional and physical offline DV victimization (verbal–emotional: β = 0.50, *p* < 0.001, physical: β = 0.18, *p* = 0.001) predicted greater cyber-control victimization.

In relation to cyber-aggression victimization (see Table 4), the proposed regression model explained 28% of variance for boys and 53% of variance for girls. For boys, the variables romantic myths (β = 0.14, *p* = 0.015) and relational and physical offline DV victimization predicted greater cyber-aggression victimization (relational: β = 0.29, *p* < 0.001, physical: β = 0.32, *p* < 0.001). For girls, the same variables were significant predictors of cyber-aggression victimization: romantic myths (β = 0.09, *p* = 0.035), relational offline DV victimization (β = 0.18, *p* = 0.003), and physical offline DV victimization (β = 0.51, *p* < 0.001).

## 4. Discussion

The purpose of this study was to analyze the relations among cyber DV victimization, offline DV victimization, and adolescents’ beliefs in myths of romantic love. Correlations among all these variables and possible differences in cyber and offline DV victimization according adolescent levels of belief in romantic myths were examined. The results showed positive correlations among the three forms of offline DV victimization (relational, verbal–emotional, and physical) and the two forms of cyber DV victimization (cyber-control and cyber-aggression) in both boys and girls. These results confirmed a close link between the real world and virtual world in adolescents [2,6,9,11,16,28,59,60], and highlighted the situation of online and offline poly-victimization that many adolescent boys and girls might find themselves in [39,42]. Positive correlations among adolescents beliefs in romantic myths and both cyber-control and cyber-aggression victimization were also observed. These positive correlations could be related to previous findings indicating that many adolescents are not aware of some aggressive and control behaviors as forms of dating violence [50,52]. Some romantic beliefs, like considering control and jealousy by partners to be signs of their love or perceiving love as suffering, could make adolescent boys and girls perceive some cyber abuse behaviors as normal behaviors in romantic relationships [14]. In the present study romantic myths were also positively related to verbal–emotional and physical offline DV victimization in adolescent girls, which indicates that romantic myths are linked not only with cyber DV victimization, but also with offline DV victimization in adolescent girls. Adolescent girls with a greater belief in romantic myths reported more cyber-control, cyber-aggression and verbal emotional offline DV victimization. Romantic myths may have a stronger impact on the offline and online behaviors that girls expect in a romantic relationship, given different socialization patterns in boys and girls. Adolescent boys and girls receive different messages about the romantic relationships from their social context [48]. Adolescent girls receive messages that make romantic love become central in developing their identity, while adolescent boys receive messages that place the public space and professional future in a more important role for developing their identity [48]. Although gender roles and stereotypes are changing in our societies, they still strongly influence adolescent boys and girls, and could make romantic myths of love more relevant for girls by increasing their risk of suffering DV victimization. Future studies should analyze this question more broadly.

As regards the role of adolescents’ beliefs in romantic myths and offline DV victimization as predictor variables of cyber DV victimization, the results confirmed the predictive weight of these variables in both boys and girls. As in previous studies, these data once again confirmed a close link between offline DV and cyber DV victimization [2,6,16,28], and also a relevant role of romantic myths in predicting cyber DV victimization in adolescents. Moreover, the results revealed some interesting differences in the main predictor variables between cyber-control victimization and cyber-aggression victimization. Thus, verbal–emotional offline DV victimization was the main predictor variable of cyber-control victimization, while physical and relational offline DV victimization were the main predictor variables of cyber-aggression in both boys and girls. Romantic myths were a positive significant predictor of cyber-control and cyber-aggression victimization in both boys and girls, although its weight better predicted cyber-control victimization in adolescent girls.

In cyber-control victimization, verbal–emotional offline DV victimization was the main predictor variable in boys and girls. Psychological/emotional offline DV involves acts like insulting, despising, humiliating, and threatening victims [33,35,38,61], and its prevalence is high in adolescent couples [30,31,32,33,34,35]. Cyber-control victimization is also more frequent in adolescents than cyber-aggression victimization [11,22,49]. The closer relations between verbal–emotional offline DV victimization and cyber-control victimization herein observed could be linked with the higher prevalence of both types of teen DV victimization and their association with adolescents’ lack of previous experience in romantic relationships [49,52,62,63,64]. Adolescents initiate their first romantic relationships in this life stage, and lack experience in handling conflicts with partners and how to express their emotions to them. They frequently believe in romantic myths that associate control and love. Adolescents’ lack of previous experience and their belief in romantic myths could increase their risk of becoming victims of both cyber-control and verbal–emotional offline DV. Romantic myths could lead adolescents to not perceive some partner behaviors like DV, which they could consider to be normal behavior in their romantic relationship. Romantic myths also highlight their perception of needing to have a partner to be happy, which could lead them to continue in a romantic relationship in which they suffer cyber-control victimization. The relevance of romantic myths in DV victimization was confirmed in the present study because this variable was a positive predictive variable of cyber-control victimization in both boys and girls.

Although myths of romantic love were a significant predictor of cyber-control victimization in both boys and girls, belief in these myths could have had a stronger impact on adolescent girls. The results indicated that the adolescent girls with greater belief in romantic myths reported not only more cyber-control victimization, but also more cyber-aggression victimization and more verbal–emotional offline DV victimization. The variance explained by romantic myths and offline DV victimization was higher for girls, and romantic myths emerged as the main predictor of cyber-control victimization in girls. Another interesting difference between boys and girls was the role played by physical offline DV victimization as a significant predictive variable of cyber-control victimization for adolescent girls, but not for adolescent boys. The adolescent girls who suffered physical offline DV victimization were also likely to suffer cyber-control victimization, but this association was not observed in adolescent boys. Physical offline DV victimization could be a higher risk factor of cyber DV victimization for girls, who would also be more likely to suffer cyber-aggression and cyber-control by their partners, and with more serious consequences for their psychosocial adjustment. These differences between boys and girls highlight the need to take a gender perspective in teen dating violence research [22,65,66], as some differences appear in the variables related to different teen DV types in adolescent boys and girls. From this gender perspective, not only should the differences between boys and girls in the relations among variables be analyzed, but also new variables, such as the extent to which girls and boys assume female and male stereotypes, their gender identity or their adhesion to male and female social norms, should be considered. Research on DV including this gender perspective could contribute to develop more effective prevention programs.

Regarding cyber-aggression victimization, the main predictor variables were physical and relational offline DV victimization for both boys and girls. The close link between physical offline DV and cyber-aggression observed in this study highlights the ease with which adolescents transfer aggression from the real world to the virtual world, and both aggression toward peers and aggression toward romantic partners [2,6,16,28,58,67,68]. Physical offline DV involves intentionally using physical force to harm victims, e.g., hitting, pushing and slapping, while cyber-aggressions includes behaviors that involve harming victims through direct attacks, e.g., threats, insults or disseminating private information [1,11,20,21,38]. Both forms of DV (cyber-aggression and offline physical aggression) involve directly harming victims and they seem closely connected. These connections should be taken into account in DV prevention programs by developing strategies to help victims to detect all the forms of aggression they may suffer offline and online [39,66]. In cyber-aggression victimization, relational offline DV victimization also appeared as a main predictor for both boys and girls. This type of offline DV involves actions like controlling what victims can and cannot do, and isolating victims from their friends and family. Some adolescents could use cyber-aggression toward partners to control their social relationships, e.g., threatening to spread humiliating messages from their partners on social networks if they continue a friendly relationship with someone whom aggressors do not want. Moreover, some of these cyber-aggression behaviors may not be perceived by adolescents as dating violence because romantic myth was also a significant predictor of cyber-aggression victimization for both boys and girls. Given the importance of romantic myths in relation to cyber-aggression and cyber-control victimization, this variable should be included in dating violence prevention programs, as previously noted [18,47], by analyzing with adolescents how these romantic myths affect to their romantic relationships.

The present study has also several limitations. First, it is a cross-sectional study, so it was not possible to conclude about any causal relations between the analyzed variables. Hence despite interesting relations being observed between offline and cyber DV victimization, adolescents could suffer both types of victimization by partners at the same time, or cyber DV victimization could even appear before offline DV victimization. Future longitudinal studies are necessary to know how some variables influence others, and to also analyze their possible mutual influences. Another limitation was the use of self-report measures, which may include some biases based on adolescents’ perceptions or the possible social desirability of their responses. Although these measure types are common in research into victimization and aggressive behavior in adolescents, it would be worthwhile using other sources of information, such as partners. It would be convenient for future studies to include an analysis of possible bidirectionality in teen DV by measuring not only DV victimization, but also DV perpetration. Moreover, some variables related to adolescents’ access to ICTs, such as their use of smartphones or how many hours a day they are connected, should be considered. It would also be interesting to use qualitative measures, such as in-depth interviews and discussion groups, to better understand how adolescents perceive their romantic relationships, the existence of some control and aggressive behaviors in these relationships, and how they consider romantic myths could influence them. Conducting quantitative and qualitative studies with larger samples, including joint samples of adolescents and youths, could provide a broad perspective that contributes to promote changes in society to prevent DV. Moreover, other variables related to the quality of their romantic relationships, such as the perceived support of the couple and their use of social networks to express their affection to partners or to increase communication with one another, could be included in future studies as possible protective factors of cyber-control and cyber-aggression victimization.

## 5. Conclusions

The present study provides interesting data on variables related to cyber DV victimization in adolescents. Adolescents’ current use of social networks to communicate and be emotionally linked with their romantic partners is high and frequently positive, but social media can also be used to perform DV. The results of the present study revealed a close association between offline DV victimization and cyber DV victimization in adolescents, which suggests that being offline-victimized by partners could be a risk factor for also being online-victimized. The results also indicated closer relations between some forms of offline DV victimization and the two analyzed forms of cyber DV victimization: cyber-control and cyber-aggression. Thus verbal–emotional offline DV victimization was the main predictor variable of cyber-control victimization, while physical and relational offline DV victimization were the main predictors of cyber-aggression. Physical offline DV victimization and cyber-aggression victimization are less frequent in adolescent couples, and their closer links herein observed could indicate more worrying DV situations with more negative consequences for adolescents. Although verbal–emotional offline DV victimization and cyber-control victimization are more frequent forms of teen dating violence and are probably more associated with adolescents’ lack of abilities and experience to initiate and maintain romantic relationships, these forms of DV victimization should also be included in prevention programs.

The intervention programs that aim to prevent DV victimization in adolescents must include strategies to help adolescents to recognize all forms of aggressive and control behaviors by partners (online and offline), like forms of dating violence, and must not be taken as normal behavior in romantic relationships. The analysis of romantic myths and how they influence adolescents’ perceptions of their romantic relationships should be made a priority objective in early intervention with adolescent boys and girls by taking into account that these myths are predictive variables of both cyber-control and cyber-aggression. Although romantic myths seem to have a stronger impact on adolescent girls, they are related to cyber-control and cyber-aggression victimization in both boys and girls. It would be useful to include these romantic beliefs in intervention programs implemented for adolescents in early adolescence, when first romantic relationships begin and adolescents create patterns of relationships that they will repeat later with other partners. In line with this, some recent programs aimed to prevent DV in adolescents include activities that focus on helping adolescents to rethink the myths of romantic love, the gender stereotypes transmitted by society (e.g., through songs, movies), the sexist attitudes (including hostile and benevolent sexism) and the attitudes of tolerance toward some forms of abuse in romantic relationships [47,69,70]. These intervention programs also encourage adolescents to develop healthy and positive romantic relationships [47,69,70]. The results of the present study provide support to prevent DV in adolescents through such prevention programs.

## Figures and Tables

**Table 1:** Pearson’s Correlations among Variables (Boys Above the Diagonal).

Variables	1	2	3	4	5	6
**1. Romantic Myths**		−0.03	0.04	0.09	0.15 *	0.16 *
**2. Relational Offline DV-V**	0.10		0.68 **	0.45 **	0.47 **	0.42 **
**3. Verbal–emotional Offline DV-V**	0.16 *	0.66 **		0.58 **	0.55**	0.39 **
**4. Physical Offline DV-V**	0.20 **	0.56 **	0.54 **		0.40**	0.46 **
**5. Cyber-control Victimization**	0.27 **	0.51 **	0.62 **	0.48 **		0.69 **
**6. Cyber-aggression Victimization**	0.23 **	0.55 **	0.53 **	0.69 **	0.54 **	

Note: DV-V = Dating Violence Victimization; ** *p* < 0.01; * *p* < 0.05

**Table 2:** Means (SD) in Offline Dating Violence Victimization and Cyber Dating Violence Victimization in Adolescent Girls and Boys with Low and High Scores for Beliefs in Romantic Myths.

**Girls**					
**Romantic Myths**					
**Variables**	**Low**	**High**	**F**	***p***	**η^2^**
**Relational Offline DV-V**	1.06 (0.21)	1.17 (0.52)	1.525	0.22	0.018
**Verbal–emotional Offline DV-V**	1.23 (0.30)	1.50 (0.59)	6.836	0.011	0.078
**Physical Offline DV-V**	1.00 (0.00)	1.12 (0.49)	2.607	0.11	0.031
**Cyber-control Victimization**	1.14 (0.20)	1.53 (0.77)	10.211	0.002	0.112
**Cyber-aggression Victimization**	1.00 (0.00)	1.17 (0.52)	4.299	0.041	0.05
**Boys**					
**Romantic Myths**					
	**Low**	**High**	**F**	***p***	**η^2^**
**Relational Offline DV-V**	1.23 (0.52)	1.25 (0.48)	0.035	0.851	0
**Verbal–emotional Offline DV-V**	1.33 (0.51)	1.47 (0.61)	1.426	0.236	0.017
**Physical Offline DV-V**	1.12 (0.45)	1.28 (0.63)	1.823	0.181	0.021
**Cyber-control Victimization**	1.43 (0.63)	1.79 (0.78)	5.374	0.023	0.062
**Cyber-aggression Victimization**	1.18 (0.54)	1.45 (0.65)	4.553	0.036	0.052

Note: SD = Standard Deviation; DV-V = Dating Violence Victimization.

**Table 3:** Linear regression analysis for predicting Cyber-control victimization.

	Boys ^1^			Girls ^2^		
	β	t	*p*	β	t	*p*
**Romantic myths**	0.14	2.539	0.012	0.16	3.352	0.001
**Relational offline DV Victimization**	0.19	2.584	0.010	0.12	1.897	0.059
**Verbal–emotional offline DV Victimization**	0.41	5.453	<0.001	0.50	9.014	<0.001
**Physical offline DV Victimization**	0.10	1.477	0.141	0.18	3.301	0.001

Note: DV = Dating Violence. ^1^ F (3, 225) = 37.83 *p* < 0.001, Adjusted R^2^ = 0.33; ^2^ F (3, 259) = 68.33 *p* < 0.001, Adjusted R^2^ = 0.43.

**Table 4:** Linear regression analysis for predicting Cyber-aggression victimization.

	Boys ^1^			Girls ^2^		
	β	t	*p*	β	t	*p*
**Romantic myths**	0.14	2.447	0.015	0.09	2.121	0.035
**Relational offline DV Victimization**	0.29	4.511	<0.001	0.18	3.025	0.003
**Verbal–emotional offline DV Victimization**	0.01	.104	0.917	0.12	2.059	0.041
**Physical offline DV Victimization**	0.32	4.959	<0.001	0.51	9.418	<0.001

Note: DV = Dating Violence. ^1^ F (3, 225) = 30.01 *p* < 0.001, Adjusted R^2^ = 0.28; ^2^ F (4, 258) = 73.79 *p* < 0.001, Adjusted R^2^ = 0.53.
